# Identification and functional analysis of key miRNAs and target genes associated with failure of HBV mother-to-child transmission prevention

**DOI:** 10.1371/journal.pone.0348899

**Published:** 2026-05-07

**Authors:** Quan He, Xiong Zou, Chunyan Zheng, Jiawei Zhang, Jialing Li, Liping Hu, Ting Zeng, Zijuan Huang, Peipei Zeng, Jinli Wei, Haichen Cui, Yongjian Su, Hai Li

**Affiliations:** 1 School of Public Health and Management, Guangxi University of Chinese Medicine, Nanning, Guangxi, China; 2 Guangxi Key Laboratory of Integrative Traditional Chinese and Western Medicine and Translational Medicine for High-Incidence Infectious Diseases, Nanning, Guangxi, China; 3 The Maternal and Child Health Hospital of Guangxi Zhuang Autonomous Region, Nanning, Guangxi, China; 4 Beihai People’s Hospital, Beihai, Guangxi, China; 5 Zhongshan Traditional Chinese Medicine Hospital, Zhongshan, Guangdong, China; 6 Guangxi Zhuang Autonomous Region Center for Disease Control and Prevention (Guangxi Zhuang Autonomous Region Academy of Preventive Medicine), Guangxi Key Laboratory for the Prevention and Control of Viral Hepatitis, Nanning, Guangxi, China; 7 Liuzhou Maternal and Child Health care Hospital, Liuzhou, Guangxi, China; 8 Tiandong County Center for Disease Control and Prevention, Baise, Guangxi, China; 9 Ruikang hospital affliated to Guangxi Univercity of Chinese Medicine, Nanning, Guangxi, China; University of Cincinnati College of Medicine, UNITED STATES OF AMERICA

## Abstract

**Background:**

Residual mother-to-child transmission (MTCT) of hepatitis B virus (HBV) remains a significant clinical challenge despite standard immunoprophylaxis. Identifying molecular markers is crucial for improved prevention and diagnosis.

**Methods:**

We conducted a case-control study using the Guangxi Liuzhou HBV MTCT registry. Peripheral blood RNA sequencing (Illumina HiSeq) was performed on infants from HBsAg-positive mothers: cases (HBsAg-positive, n = 6) and controls (HBsAg-negative, n = 10). All infants receive HBIG and the first dose of hepatitis B vaccine within 24 hours after birth, followed by completion of the three-dose vaccination series. Differentially expressed miRNAs (DEMs; adj-p < 0.05, |log2FC| > 1) were identified. Target genes were predicted (miRanda/RNAhybrid) and functionally analyzed (GO/KEGG enrichment, PPI network). HBV-associated target genes were identified by cross-referencing GeneCards/NCBI.

**Results:**

RNA-seq identified 62 DEMs (19 upregulated, 43 downregulated). Target prediction yielded 5,014 genes. Functional enrichment highlighted key pathways and processes. PPI analysis pinpointed highly connected genes. Integration with HBV databases revealed 3 key target genes potentially modulated by 4 specific DEMs (hsa-miR-6747-3p, hsa-miR-4772-3p upregulated; hsa-miR-4676-5p, hsa-miR-485-5p downregulated).

**Conclusion:**

This study identifies dysregulation of 4 key miRNAs and their association with 3 HBV-linked target genes as potential contributors to residual HBV MTCT. These findings provide novel insights into the molecular mechanisms underlying HBV MTCT and suggest potential targets for intervention.

## 1. Introduction

Hepatitis B virus (HBV) infection remains a major global public health challenge. As a highly transmissible pathogen, HBV can lead to chronic hepatitis, liver cirrhosis, and hepatocellular carcinoma, resulting in substantial morbidity and mortality worldwide. According to estimates by the World Health Organization [1], approximately 254 million people were living with chronic HBV infection globally as of 2022, and about 1.1 million deaths occur each year due to HBV-related liver diseases, including cirrhosis and hepatocellular carcinoma. Despite the availability of effective vaccines and antiviral therapies, HBV continues to impose a considerable health burden, particularly in low- and middle-income countries, where marked geographic disparities in prevalence and disease outcomes persist. Although effective vaccination, diagnosis, and antiviral treatment can substantially reduce the disease burden, insufficient coverage remains a major barrier, and the global prevention and control of HBV still face significant challenges [2].

China is one of the countries with the highest HBV burden worldwide. National epidemiological data indicate that approximately 75 million people are currently infected with HBV in China, including about 30 million individuals with chronic hepatitis B, accounting for nearly one-third of the global population living with chronic HBV infection [3,4]. Although expanded vaccination programs and prevention strategies have markedly reduced HBV infection rates among children in recent decades, the prevalence of chronic HBV infection remains high in the adult population. Chronic HBV infection continues to be a leading cause of liver-related mortality in China, with an estimated hundreds of thousands of deaths annually attributable to HBV-associated liver diseases. Owing to the extremely low rate of functional cure, current antiviral therapies are effective in suppressing viral replication but result in hepatitis B surface antigen (HBsAg) loss in only a small proportion of patients each year [3]. The large population size, high population mobility, and prolonged disease course collectively contribute to the sustained public health burden of HBV in China.

HBV is primarily transmitted through blood exposure, sexual contact, and mother-to-child transmission (MTCT). In regions with high endemicity, particularly China, MTCT represents a dominant route of infection and a key driver of chronic HBV persistence. With the successful implementation of universal infant hepatitis B vaccination programs, horizontal transmission has been markedly reduced [5–7]. Consequently, MTCT has emerged as the principal source of new chronic HBV infections in China. Although combined immunoprophylaxis strategies—including maternal antiviral therapy during pregnancy, timely neonatal vaccination, and hepatitis B immunoglobulin administration—have substantially lowered MTCT rates, a small proportion of infants still acquire HBV infection despite standard preventive measures. This phenomenon is more appropriately described as breakthrough or residual MTCT rather than “failure of MTCT blockade.” Previous studies have identified several maternal and placental risk factors associated with residual MTCT, including maternal HBeAg positivity, high maternal serum HBV DNA levels (>10⁶ IU/mL), placental HBV antigen expression, PreS1 positivity, maternal overweight status, and disease severity [8,9]. However, the molecular mechanisms underlying residual MTCT remain poorly understood.

MicroRNAs (miRNAs) are a class of endogenous, non-coding RNAs approximately 21–23 nucleotides in length that regulate gene expression at the post-transcriptional level by binding to complementary sequences in target messenger RNAs. Although miRNAs do not encode proteins, they play critical roles in diverse biological processes, including cell differentiation, immune regulation, and viral infection. A single miRNA can regulate multiple target genes, thereby exerting pleiotropic biological effects [10]. Increasing evidence suggests that miRNAs are involved in the pathogenesis of acute and chronic liver diseases, including viral hepatitis [11]. Previous studies have reported several miRNAs implicated in HBV pathogenesis, such as miR-122, miR-125a-5p, miR-199a-3p, and miR-155, which may affect viral replication, host immune responses, and inflammation signaling [12–15]. Dysregulated miRNA expression has been implicated in HBV replication, liver inflammation, fibrosis, and hepatocarcinogenesis, potentially through modulation of host immune responses, viral entry receptors, and hormone-related signaling pathways [16]. However, to date, the role of miRNAs in HBV mother-to-child transmission has not been systematically investigated.

We hypothesize that specific miRNA expression profiles in maternal or placental tissues are associated with residual HBV MTCT despite standard immunoprophylaxis, and that these miRNAs may regulate key genes involved in viral transmission or host defense mechanisms. Therefore, the aim of this study was to identify miRNAs associated with the failure of HBV mother-to-child transmission and to predict their potential target genes, providing molecular insights for early intervention. Specifically, this study will employ high-throughput sequencing, differential expression analysis (DESeq2), miRanda/RNA hybrid target prediction, Gene Ontology (GO) and Kyoto Encyclopedia of Genes and Genomes (KEGG) enrichment analyses, protein–protein interaction (PPI) network construction, and data mining from GeneCards and NCBI databases to identify differentially expressed miRNAs and their target genes associated with residual HBV mother-to-child transmission (MTCT). These findings are expected to provide novel molecular insights into HBV MTCT and lay a theoretical foundation for early risk prediction and targeted preventive strategies.

## 2. Materials and methods

### 2.1 Study subjects

This study employed a non-random sampling method to enroll infants born to hepatitis B surface antigen (HBsAg)-positive mothers. These mothers were registered at hospitals and community health service centers in Liuzhou City, Guangxi Zhuang Autonomous Region, and also recorded in the “Liuzhou Hepatitis B Mother-to-Child Transmission Information Management System” from July 2023 to January 2024. The recruitment period for this study is from 1/7/2023–31/1/2024. Follow-up assessments were conducted for the infants and their mothers when the infants were 7–24 months old. Collected data included maternal demographic characteristics, natal examination records, serological test results of HBsAg during pregnancy, as well as infant demographic characteristics, birth information, and hepatitis B vaccination status. Based on the serological test results obtained during the follow-up, infants born to HBsAg-positive mothers who themselves tested positive for HBsAg were classified into the case group. In contrast, infants born to HBsAg-positive mothers who tested negative for HBsAg at 7–24 months of age were assigned to the healthy control group.

This study was approved by the Ethics Committee of Guangxi University of Chinese Medicine, and written informed consent was obtained directly from all participating mothers (Approval No.: GXUCM IRB H 2023−06).

### 2.2 Inclusion and exclusion criteria

#### 2.2.1 Inclusion criteria.

**Mothers.**

1) Fulfilled the diagnostic criteria for chronic hepatitis B according to the “Guidelines for the Prevention and Treatment of Chronic Hepatitis B (2022 Edition) [17].
2) Tested positive for HBsAg during pregnancy.
3) Had no history of severe brain, heart, liver, or kidney diseases.

**Infants.**

1) Aged between 7 and 24 months.
2) Had received at least three doses of hepatitis B vaccine, with the last dose administered at least one month prior to enrollment.
3) Had no history of severe brain, heart, liver, or kidney diseases and were in suitable health for blood collection.

#### 2.2.2 Exclusion criteria.

**Mothers.**

1) Had co-infection with other pathogens during pregnancy (e.g., HAV, HCV, HDV, HEV, HIV, syphilis, toxoplasmosis, rubella virus, cytomegalovirus, herpes simplex virus).
2) Declined to participate in follow-up for themselves or their infants.

**Infants.**

1) Had a history of blood transfusion.
2) Had insufficient blood samples for HBV serological marker testing.
3) Had legal guardians who declined their participation in follow-up.

### 2.3 Sample size calculation

The statistical power was calculated using the RNASeqPower package by Hart et al. [18]. For fixed intra-group variance (default package settings), statistical power increases with effect size, sequencing depth, and the number of replicates per group. The table displays the statistical power for genes with 70 aligned reads, which corresponds to the median coverage of protein-coding genes in whole blood RNA-seq samples from the GTEx project (30 million aligned reads). According to the tabulated data, a sample size of 5 achieved an effect size of 98%. In this study, the case group comprised 6 sample and the control group 10 samples, yielding an effect size exceeding 98% with high reliability.

### 2.4 HBV DNA detection method

Real-time quantitative PCR (RT-qPCR) was performed with a linear range of 20–500 IU/mL, where the lower limit of detection (LOD) was 20 IU/mL and the upper limit of quantification (ULOQ) was 500 IU/mL. A measured value of 5.00 × 10² IU/mL (5.00E + 02) corresponds to 500 IU/mL. Values reported as <5.00 × 10¹ IU/mL (<5.00E + 01) indicate either HBV DNA levels below the LOD (20 IU/mL) or absence of detectable HBV DNA.

### 2.5 Extraction and quality inspection of total RNA

Whole blood samples were collected in PAXgene Blood RNA Tubes (cat. no. 762165; hereafter referred to as PAXgene tubes). Total RNA was manually extracted and purified using the PAXgene Blood RNA Kit according to the manufacturer’s instructions.

RNA concentration was quantified using a Qubit fluorometer. RNA purity was assessed using a NanoDrop spectrophotometer by measuring OD260/280 (acceptable range: 1.8–2.2) and OD260/230 (≥ 2.0). RNA integrity was evaluated by agarose gel electrophoresis and further assessed using an Agilent 2100 Bioanalyzer to determine the RNA integrity number (RIN). Samples were graded based on both RNA quantity and quality, with the final classification determined by the lower of the two ratings. Samples were classified into four quality grades based on RNA yield, concentration, integrity, and DNA contamination. Grade A samples met the following criteria: total RNA ≥ 4 μg, concentration ≥ 100 ng/μL, RNA integrity number (RIN) ≥ 7, and no detectable DNA contamination. Grade B samples had a total RNA amount ≥ 2 μg with a concentration ≥ 100 ng/μL. Grade C samples had a total RNA amount ≥ 1 μg with a concentration ≥ 50 ng/μL and RIN ≥ 6.5, or exhibited minor DNA contamination. Grade D samples had a total RNA amount < 1 μg, concentration < 50 ng/μL, RIN < 6.5, or showed substantial DNA contamination. Only Grade A and Grade B samples were considered to pass quality control and were used for subsequent sequencing library construction, whereas Grade C and Grade D samples were excluded or replaced.

### 2.6 Bioinformatics analysis

#### 2.6.1 Identification of differentially expressed miRNAs (DEmiRNAs).

The total RNA extracted from the two groups of children was sequenced using high-throughput sequencing technology.The high-throughput sequencing standard for miRNA libraries requires an RNA sample concentration of ≥ 200 ng/µ L, a total amount of ≥ 2 μ g, and an OD260/280 value between 1.8 and 2.2, OD260/230 ≥ 2.0, And the RIN detected by Agilent 2100 Bioanalyzer is ≥ 7. Using Illumina high-throughput sequencing platform and 2 × 150 bp double ended sequencing strategy to sequence the library.Based on the sequencing results, raw data were uploaded to the Genesky Biotech Tianhao Cloud Platform (http://cloud.geneskybiotech.com/#/tools/all) for analysis. This platform generated miRNA expression profiles for both the case group and control group. The following visualizations were created: Scatter plot of differentially expressed miRNAs; M-A plot (intensity ratio vs. mean intensity); Volcano plot (top 5 significant miRNAs); Hierarchical clustering heatmap (top 50 miRNAs)

Differential expression analysis was performed using DESeq2 [19] (Differentially Expressed Genes analysis package). P-adj were calculated, and miRNAs were classified as follows: Non-significant (Not DEG): P-adj > 0.05 and |log2(fold change)| ≤ 1; Differentially Expressed (DEG): P-adj < 0.05 and |log2(fold change)| > 1

#### 2.6.2 Prediction and screening of target genes for differentially expressed miRNAs (DEmiRNAs).

miRNAs regulate gene expression by binding to specific mRNA targets, collectively termed the miRNA’s target genes (or targets). Based on the DEmiRNAs identified in previous steps, target gene prediction was performed by analyzing two criteria: (1) complementarity between the miRNA seed region and the 3’ untranslated region (3’ UTR) of the target mRNA; and (2) the binding free energy of the miRNA-mRNA interaction. In this study, two computational tools were employed for miRNA target prediction: miRanda [20] (a microRNA target prediction algorithm) and RNAhybrid [21] (an energy-based miRNA-mRNA binding analysis tool).

#### 2.6.3 GO and KEGG enrichment analysis of target genes.

Using the clusterProfiler package in R(version 4.3.1) [22,23], GO and KEGG enrichment analyses were performed on the selected target genes of differentially expressed miRNAs. Visualizations were generated using the ggplot2 package in R(version 4.3.1). Target genes enriched in the top 10 most significant GO functional categories (P-adj < 0.05) were retained for further analysis. Similarly, target genes enriched in the top 10 most significant KEGG pathways (P-adj < 0.05) were identified.

### 2.7 Protein-protein interaction network analysis of target genes

Using the STRING database, a PPI network analysis was performed on the target genes selected through GO and KEGG enrichment screening. The interaction data generated by STRING were imported into Cytoscape software for visualization and extraction of highly interconnected genes, which were then used to construct the PPI network.

### 2.8 Screening of public databases

Genes associated with HBV infection and immune regulation were retrieved from the NCBI Gene Database and GeneCards Database. These genes were cross-referenced with the differentially expressed genes identified in this study to obtain the intersection. The overlapping genes were defined as the final target genes linked to HBV MTCT blockade failure and their associated miRNAs.

## 3. Results

### 3.1 Baseline characteristics of participants

**Fig 1:**
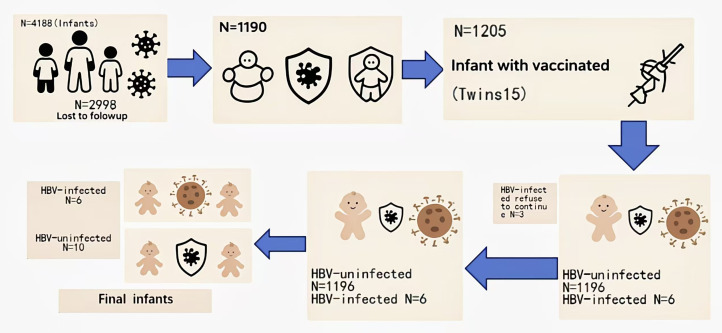
Selection process.

This study initially enrolled 4188 infants born to HBsAg-positive mothers for follow-up. Successfully followed were 1,205 infants (including 15 twin pairs) from 1,190 HBsAg-positive mothers. Based on the outcomes of HBV mother-to-child transmission (MTCT) prevention strategies, infants from 9 HBsAg-positive mothers were initially identified as HBV-positive and assigned to the case group. However, three families declined to participate, resulting in a final case cohort of 6 infants. For transcriptome sequencing analysis, 10 infants born to HBsAg-positive mothers were randomly selected as the healthy control group, matched by weight and height. The detailed participant selection process is illustrated in Fig 1.

Mothers in both the case and control groups exhibited HBeAg-positive chronic HBV infection during prenatal examinations. All infants were normal birth weight and full-term at delivery. They had completed the 3-dose hepatitis B vaccine (HepB) series (active immunization) and received a single 100-IU dose of hepatitis B immunoglobulin (HBIG) (passive immunization) within 24 hours of birth.

The residual HBV MTCT group comprised 3 males and 3 females, whereas the non-transmission group included 5 males and 5 females.

Maternal age at delivery, mode of delivery, and infant follow-up age are presented in Table 1. All six mothers in the residual transmission group had high prenatal HBV DNA levels, while five of ten mothers in the non-transmission group had high HBV DNA levels. Information on maternal antiviral therapy during pregnancy is also summarized in Table 1.

**Table 1:** The basic situation of the two groups was compared.

Variable	Case Group	Control Group
	*n* = 6	*n* = 10
**Maternal age**	31.67 ± 2.35	31.14 ± 4.7
**HBV DNA test for maternal prenatal examination (*n,%*)**		
≥ 2 × 10^5^ IU/mL	6 (100)	6 (60)
< 2 × 10^5^ IU/mL	0 (0)	4 (40)
**Maternal antiviral treatment (*n,%*)**		
No medication was used	4 (60)	5 (50)
Medication	2 (40)	5 (50)
**Mode of delivery (*n,%*)**		
Natural childbirth	4 (66.67)	8 (80)
Emergency cesarean section	1 (16.67)	1 (10)
Choose a cesarean section	1 (16.67)	1 (10)
**Infant age at follow-up**	20.67 ± 3.68	11.86 ± 0.35

### 3.2 Screening of differentially expressed miRNAs

**Fig 2:**
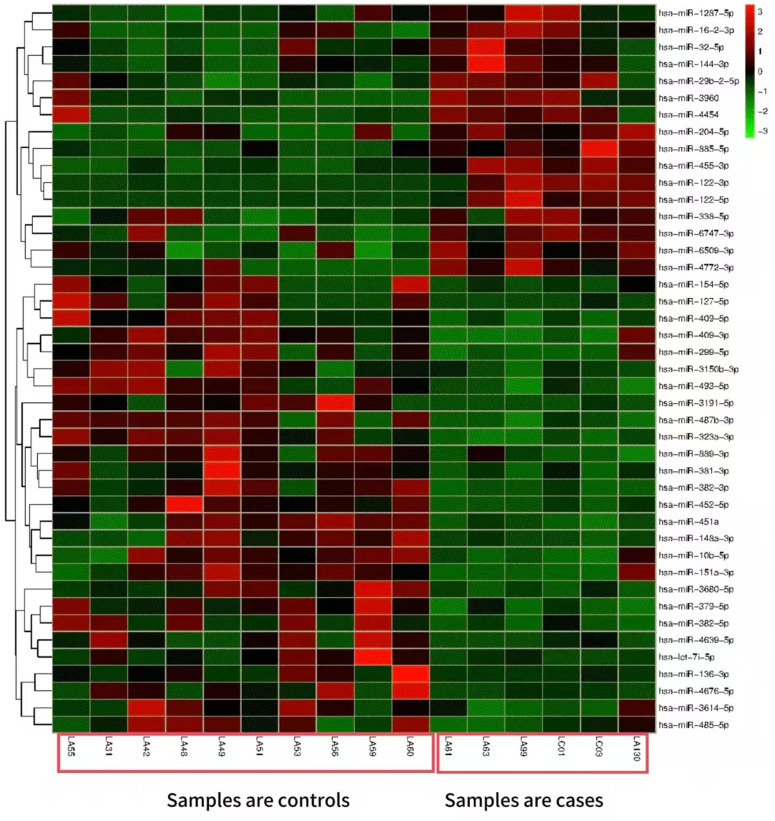
Volcano diagram of differentially expressed miRNA expression (top50).

**Fig 3:**
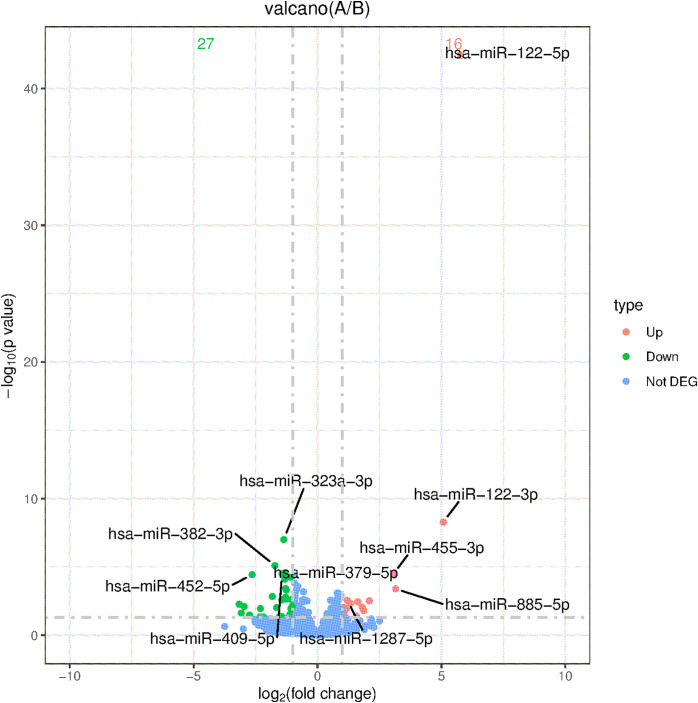
Differentially expressed miRNA clustering heatmap (top 5).

Using screening criteria (P-adj < 0.05 and |log2(fold change)| > 1), 19 upregulated miRNAs (higher expression in the case group) and 43 downregulated miRNAs (lower expression in the case group) were identified. The most significantly upregulated miRNA was hsa-miR-122-5p (log2FoldChange = 5.77), while the most downregulated was hsa-miR-323a-3p (log2FoldChange = 40.08). Results were visualized as a volcano plot (top 50 miRNAs, Fig 2), and a clustered heatmap (top 5 miRNAs, Fig 3).

### 3.3 Prediction and screening of miRNA target genes

Target genes of differentially expressed miRNAs were predicted using two algorithms, miRanda and RNAhybrid, with the intersection of both methods identifying 5,014 candidate targets.

### 3.4 GO and KEGG enrichment analysis of target genes

#### 3.4.1 GO functional enrichment.

**Fig 4:**
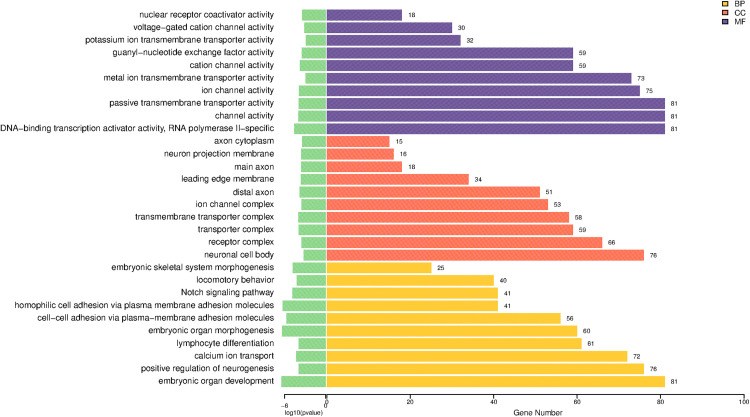
GO functional enrichment bar chart of differentially differentiated genes.

GO enrichment analysis was performed using the clusterProfiler package in R. A total of 709 biological processes (BP), 109 cellular components (CC), and 91 molecular functions (MF) were significantly enriched. The top 10 enriched terms from each category (BP, CC, MF) were visualized in a bar plot (Fig 4).

The GO Functional Enrichment Results demonstrated that the predicted target genes were primarily involved in biological processes such as embryonic organ development, embryonic organ morphogenesis, homophilic cell adhesion via plasma membrane adhesion molecules, cell-cell adhesion via plasma membrane adhesion molecules, and the Notch signaling pathway. These genes were enriched in cellular components including transmembrane transporter complexes, transporter complexes, distal axons, leading-edge membranes, and primary axon components. Molecular functions significantly associated with these genes included DNA-binding transcription activator activity, RNA polymerase II specificity, channel activity, passive transmembrane transporter activity, ion channel activity, and cation channel activity. Based on the top 10 enriched terms, 489 target genes regulated by differentially expressed miRNAs were identified.

#### 3.4.2 KEGG pathway enrichment analysis.

**Fig 5:**
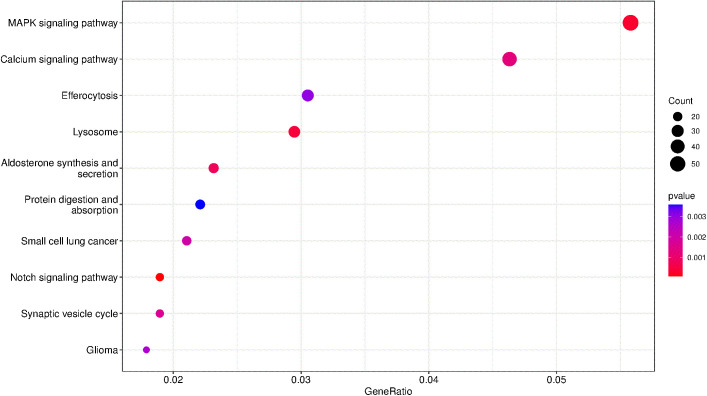
KEGG pathway enrichment scatter map (top10).

KEGG pathway enrichment analysis was conducted using the clusterProfiler package in R. A total of 59 pathways were significantly enriched, primarily including the Notch signaling pathway, MAPK signaling pathway, lysosome pathway, aldosterone synthesis and secretion, and calcium signaling pathway. The top 10 most significantly enriched pathways were visualized in a scatter plot (Fig 5).

Based on these top 10 enriched pathways, 99 target genes regulated by differentially expressed miRNAs were identified.

#### 3.4.3 Integrated GO and KEGG analysis.

Intersection analysis of genes enriched in the top 10 GO terms and top 10 KEGG pathways identified 55 target genes regulated by differentially expressed miRNAs.

### 3.5 PPI analysis of target genes

**Fig 6:**
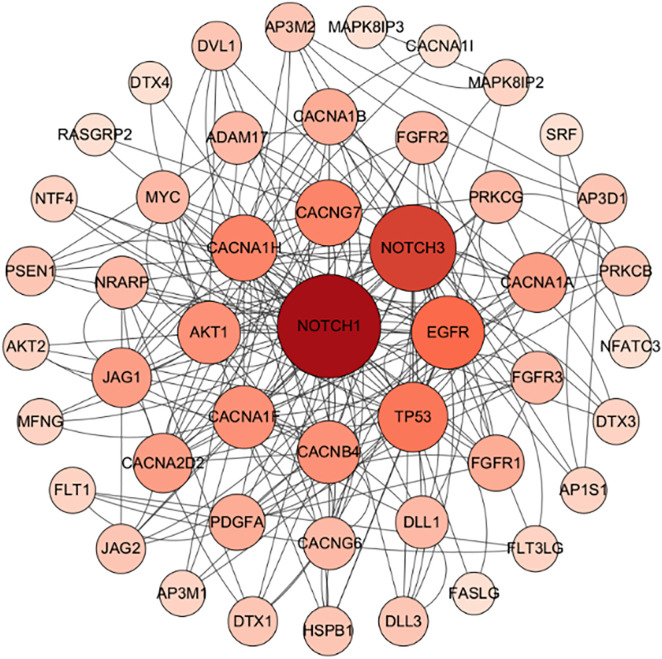
PPI plot of protein-protein interaction of the target genes.

The proteins encoded by the target genes exhibited complex interaction networks. PPI network analysis was performed using the STRING database for genes identified through GO and KEGG enrichment analyses, The STRING database was used with a confidence score threshold of 0.7, and high-confidence interactions were selected to construct the PPI network. Network hub genes were identified in Cytoscape based on degree centrality. Results from the STRING database were imported into Cytoscape for advanced network visualization. Five target genes—NOTCH1, NOTCH3, EGFR, TP53, and CACNA1H—demonstrated the highest connectivity with other genes in the network (Fig 6), suggesting their encoded proteins may broadly regulate the expression levels of other gene products.

### 3.6 Screening of public databases

Using the search terms “HBV infection or immunoregulation”, 3,108 genes were retrieved from the GeneCards database and 523 genes from the NCBI Gene database. Cross-referencing the target genes identified in the aforementioned PPI analysis with these databases revealed three overlapping genes (NOTCH1, EGFR, TP53). Further analysis of the predicted target genes identified three differentially expressed miRNAs regulating these overlapping genes.

NOTCH1: The Upregulation of hsa-miR-6747-3p can affect the blockade of MTCT of HBV by directing the target gene NOTCH1 through transcript ENST00000651671.1, ENST00000680133.1, ENST00000680218.1, ENST00000680668.1, ENST00000680778.1.

EGFR: The downregulation of hsa-miR-4676-5p can affect the blockade of MTCT of HBV by directing the target gene EGFR through transcript ENST00000455089.5

TP53: The downregulation of hsa-miR-485-5p can affect the blockade of HBV in mothers and infants by directing the target gene TP53 through transcript ENST00000576024.1. Upregulation of hsa-miR-4772-3p can affect the blockade of MTCT of HBV by directing the target gene TP53 through transcript ENST00000359597.8.

## 4. Discussion

Currently, intrapartum transmission remains a critical route for HBV spread from mother to child. Preventing MTCT is pivotal for reducing HBV infection rates. Current strategies primarily involve administering hepatitis B immunoglobulin and the hepatitis B vaccine to newborns [24]. However, HBV prophylaxis failure persists, with a reported failure rate of 4.87% [25]. Due to their immature immune systems and lower immune resistance compared to adults, HBV infection in infants often leads to more severe clinical outcomes. Emerging studies highlight that miRNAs, while not directly encoding proteins, regulate mRNA through post-transcriptional mechanisms, influencing protein translation, modification, and ultimately cellular or organism-level functions [26].

This study analyzed peripheral blood samples from infants with successful or failed blockade of HBV MTCT. High-throughput whole transcriptome sequencing identified 62 differentially expressed miRNAs, including 43 upregulated and 19 downregulated miRNAs in the case group. Target gene prediction for these miRNAs yielded 5014 candidate genes. GO enrichment analysis revealed 489 genes enriched in the top 10 biological pathways, while KEGG pathway analysis identified 99 genes enriched in the top 10 pathways. Intersection analysis of GO and KEGG results prioritized 55 overlapping target genes. Protein-protein interaction network analysis of 55 candidate genes highlighted 5 genes with the highest connectivity. Cross-referencing these 5 genes with HBV infection- related and immune-related genes from the GeneCards and NCBI Gene databases identified 3 key regulatory target genes.

Importantly, despite the small sample size, all mothers in the case group exhibited high HBV DNA levels, which did not reach statistical significance when compared to the control group (>0.05). However, from a clinical perspective, the 100% high viral load in mothers of the failure group remains highly relevant. This finding underscores that high maternal HBV viral load is a critical risk factor for MTCT blockade failure, suggesting that clinical management should prioritize monitoring and controlling maternal viral load, even when statistical differences are not observed due to limited sample size.

The Notch signaling pathway is one of the most frequently activated pathways in human liver diseases. Previous studies have demonstrated that NOTCH1 plays a critical role in immune regulation by modulating the development and function of B and T lymphocytes and enhancing antibody secretion following B-cell activation [27]. Further evidence indicates that Notch1-mediated signaling negatively regulates NLRP3 inflammasome–driven innate immune responses during hepatitis and exerts broad immunomodulatory effects by inducing regulatory T cells (Tregs) [28]. In addition, NOTCH1 is involved in the regulation of hepatic inflammation and tissue injury, and has been identified as a key mediator in liver ischemia–reperfusion injury and inflammatory cascades [29,30]. In the present study, we identified upregulation of hsa-miR-6747-3p targeting NOTCH1. Dysregulation of this miRNA–gene axis may interfere with early-life immune regulation and inflammatory balance in infants, thereby indirectly impairing antiviral immune responses and increasing the risk of failure in preventing mother-to-child transmission of HBV.

The epidermal growth factor receptor (EGFR) is a central regulator of cell proliferation, differentiation, and migration, and plays a pivotal role in hepatocyte regeneration, tissue repair, and inflammatory modulation [31–33]. Accumulating evidence suggests that EGFR signaling contributes not only to liver homeostasis but also to the regulation of pro-inflammatory responses and cell survival pathways during hepatic injury [34,35]. Although EGFR is not a classical HBV-specific immune gene, its involvement in hepatocyte signaling networks and virus entry–associated pathways has increasingly been recognized. In this study, downregulation of hsa-miR-4676-5p was predicted to target EGFR, potentially leading to dysregulated EGFR expression. Such alterations may compromise hepatocyte regenerative capacity and inflammatory control, thereby creating a permissive host environment for persistent perinatal HBV infection and failure of mother-to-child transmission prevention.

TP53 is a well-established tumor suppressor gene that regulates a wide range of cellular processes, including cell cycle arrest, apoptosis, DNA repair, autophagy, and metabolic homeostasis [36,37]. Beyond its canonical role in tumor suppression, emerging studies indicate that TP53 also contributes to host immune regulation by influencing the recruitment and functional activity of bone marrow–derived immune cells and T lymphocytes [38]. Although the role of TP53 in HBV mother-to-child transmission has not been directly elucidated, its involvement in antiviral immune responses and hepatocyte stress signaling suggests that aberrant TP53 expression may indirectly modulate host–virus interactions. Consequently, TP53 was retained as a candidate key target gene in this study. Nevertheless, we emphasize that its potential role in HBV mother-to-child transmission Prevention failure requires further functional validation.

Despite the insights provided by this study, several limitations should be acknowledged. First, the relatively small sample size and exploratory design may limit the generalizability of the findings and reduce statistical power to detect intergroup differences. Second, the use of non-random sampling may have introduced selection bias. Third, complete maternal HBV genotype data were unavailable for a subset of participants, precluding fully matched or stratified analyses by maternal genotype and thereby potentially limiting the evaluation of genotype-specific effects on HBV mother-to-child transmission (MTCT). Fourth, although candidate miRNAs and their predicted target genes were identified through computational analyses, functional validation experiments were not performed. Accordingly, the mechanistic roles of these miRNAs and their targets in blocking HBV MTCT remain to be clarified in future experimental studies.

Importantly, given the limited sample size and exploratory nature of the present work, validation in larger, multi-center cohorts is warranted. The establishment of a coordinated registry for residual HBV MTCT cases could facilitate the systematic collection of biological specimens and standardized clinical data, thereby enabling robust validation of candidate miRNAs and the identification of additional biomarkers. Such collaborative and well-powered investigations would not only strengthen the clinical relevance and mechanistic interpretation of miRNA-mediated regulation in HBV MTCT, but also provide a framework for studying other incident HBV infections, including horizontal transmission in diverse populations.

## 5. Conclusions

This study identified four candidate miRNAs (hsa-miR-6747-3p, hsa-miR-4676-5p, hsa-miR-485-5p, and hsa-miR-4772-3p) and their potential target genes (NOTCH1, EGFR, and TP53) associated with the prevention of mother-to-child transmission of hepatitis B virus. Dysregulation of these miRNAs may influence immune responses to HBV infection and viral replication by modulating key pathways involved in immune signaling, hepatocyte biology, and cellular stress and repair processes, thereby contributing to the regulation of HBV MTCT. Overall, this study highlights the potential biological roles of these candidate miRNAs in HBV MTCT at the pathway and functional levels; however, their precise molecular mechanisms require further validation through in vitro and in vivo functional experiments.
